# Incidence, sociodemographic and presenting clinical features of childhood non-infectious uveitis: findings from the UK national inception cohort study

**DOI:** 10.1136/bjo-2024-326674

**Published:** 2025-02-11

**Authors:** Ameenat Lola Solebo, Salomey Kellett, Eibhlin McLoone, Harry Petrushkin, Jose Gonzalez-Martin, Jane Ashworth, Jessy Choi, Rachel F Pilling, Kate Armon, Kishore Warrier, Srilakshmi M Sharma, Jugnoo S Rahi, Members of the UNICORNS (Uveitis in Childhood National Cohort Study) Group, Jose Gonzalez-Martin

**Affiliations:** 1 Population Policy and Practice Research and Teaching Department, UCL GOS Institute of Child Health, London, UK; 2 Great Ormond Street Hospital for Children NHS Trust, London, UK; 3 UCL GOS Institute of Child Health, London, UK; 4 Ophthalmology, Royal Victoria Hospital, Belfast, UK; 5 Uveitis and Scleritis Service, Moorfields Eye Hospital NHS Foundation Trust, London, London, UK; 6 Rheumatology, Great Ormond Street Hospital for Children, London, UK; 7 Paediatric Ophthalmology, Alder Hey Children's NHS Foundation Trust, Liverpool, Merseyside, UK; 8 Manchester Royal Eye Hospital, Manchester University Hospitals NHS Trust, Manchester, UK; 9 Sheffield Children's Hospital NHS Foundation Trust, Sheffield, UK; 10 Ophthalmology Department, Bradford Royal Infirmary, Bradford, West Yorkshire, UK; 11 Cambridge University Hospitals NHS Foundation Trust, Cambridge, UK; 12 Nottingham University Hospitals NHS Trust, Nottingham, UK; 13 Oxford Eye Hospital, John Radcliffe Hospital, Oxford, UK; 14 Population, Policy and Practice Research and Teaching Department, UCL Great Ormond Street Institute of Child Health Population Policy and Practice, London, UK

**Keywords:** Uveitis, Child health (paediatrics), Epidemiology

## Abstract

**Background:**

We aimed to provide, through the Uveitis in Childhood National Cohort Study, population-based evidence on incidence, distribution and disease characteristics for childhood onset non-infectious uveitis.

**Methods:**

Eligible children and young people (<18 years) were those newly diagnosed with non-infectious uveitis between 1 March 2020 and 28 February 2023. Cases were identified and recruited through passive surveillance across a multicentre network. Descriptive analysis of demographic, socioeconomic and clinical characteristics at diagnosis is reported alongside incidence rates, relative rates by region and sociodemographic patterning.

**Results:**

468 cases were identified, providing a minimal national disease incidence of 1.89/100 000 (95% CI 1.72 to 2.07). Among the 255 children recruited, anterior uveitis was predominant (76.9%) and 65% of cases were bilateral. Peak incidence was at 11–15 years. Children resident in deprived areas and those from non-White ethnic backgrounds were over-represented (28% and 31% of the cohort). One in seven children (15%) had a diagnosis of juvenile idiopathic arthritis (JIA), and 5% had tubulointerstitial nephritis. Although bilaterally poor vision was uncommon (16.8%), 44.3% had lost some vision in at least one eye.

**Conclusions:**

There is a need to reconsider how best to deliver paediatric rheumatological and eye care that meets the needs of young people, as well as young children, with uveitis. The predominance of non-JIA-related uveitis calls for a shift in focus. There appears to be socioeconomic drivers of disease risk, which are worthy of future exploration and which have implications on the delivery of care for this chronic and blinding disease.

**WHAT IS ALREADY KNOWN ON THIS TOPIC**

- Childhood uveitis is a rare but impactful chronic disorder with recognised associations with systemic disorders such as juvenile idiopathic arthritis (JIA).

**WHAT THIS STUDY ADDS**

- Disease incidence is higher than expected, particularly among children living in relative socioeconomic deprivation or from ethnic minority backgrounds. Non-JIA-associated disease is prevalent (85% of cases) at uveitis diagnosis, and adolescence is the period of greatest risk of disease onset. Almost half of the cohort had some degree of vision loss at diagnosis.

**HOW THIS STUDY MIGHT AFFECT RESEARCH, PRACTICE OR POLICY**

- There is a need to reconsider how best to deliver timely, multidisciplinary care structures that meet the needs of young people, as well as young children, with uveitis.

## Introduction

 Childhood onset uveitis, a descriptive term for eye disorders unified by intraocular inflammation, is uncommon but impactful, conferring a life-long risk of visual impairment,^1^ with attendant negative impact on broader development, education, social opportunities and quality of life.^2^ Additional non-ocular immune mediated disorders often co-exist or manifest with uveitis.^3^ Juvenile idiopathic arthritis (JIA)-associated uveitis has been considered the most common form of disease.^1 3 4^

Children diagnosed with uveitis typically require treatment with disease-modifying anti-rheumatic drugs (DMARDs) to prevent permanent visual loss. Home administration of oral or subcutaneous DMARDs and high-cost monoclonal antibody therapies have supported improved outcomes.^5^ However, care may involve the use of agents which may require frequent hospital administration, with high healthcare resource use and significant opportunity and financial costs for the affected child (eg, time away from school) and their families (eg, time away from work).^2^ Care is multidisciplinary. This meets the needs generated by co-occurrent multisystem inflammatory disorders and the needs generated by the use of immunomodulatory therapies in children with ‘isolated’ uveitis.^6^

Commissioning and implementation of equitable services and evaluation of effectiveness of healthcare requires understanding of incidence and determinants of disease risk. Previous estimates of incidence of childhood uveitis in the UK have been drawn from regional studies^4 7^ or have been restricted to disease subsets such as those with uveitis associated with JIA.^8^ Furthermore, disease incidence may have changed. First, it is due to the increasing prevalence of other immune-mediated disease,^9^ particularly during and following the global COVID-19 pandemic.^10^ Second, it is due to changing incidence of specific forms of uveitis, for example, a reduction in JIA-associated uveitis through improved care for that population.^11^ We report current demographic, socioeconomic and clinical characteristics at diagnosis of childhood onset non-infectious uveitis together with overall disease incidence and geographic and sociodemographic variations, drawing on the UNICORNS or the Uveitis in Childhood National Cohort Study.

## Patients and methods

### Study design

UNICORNS is a UK-wide prospective inception longitudinal study established in 2020 in collaboration with a multicentre clinical research network representing all four nation states of the UK.^12^

### Case definition/eligibility criteria

Any child/young person aged <18 years resident in the UK and newly diagnosed with any form of non-infectious uveitis.

### Case identification

Study methods have been described in detail elsewhere.^12^ In brief, cases were identified using passive surveillance between 1 March 2020 and 28 February 2023, undertaken through a multicentre clinical collaborative network, the Paediatric Ocular Inflammation Group (POIG). POIG comprises 68 paediatric ophthalmologists, rheumatologists and other consultant (‘attending’) medical specialists managing children with ocular inflammatory disorders, including representation of all of the 27 children’s hospitals and eight eye hospitals across the UK.^12^

### Data collection and management

Clinical and demographic data were collected from collaborating clinical sites using a secure REDCap database.^12 13^ These were reviewed for completeness by a senior ophthalmologist (ALS), and reporting clinicians were contacted about any incomplete or inconsistent data. Families were also asked to self-report ethnicity.

### Analysis

Children were grouped by uveitis anatomical location (anterior, anterior and intermediate, intermediate, posterior or panuveitis) using the definitions established by the Standardisation of Uveitis Nomenclature group.^14^ Patient socioeconomic status was categorised using the UK Index of Multiple Deprivation (IMD), derived from family residence postal (zip) code^15^ and grouped into quintile rankings. Ethnicity was categorised using the UK Office of National Statistics (ONS) coding.^16^

Disease severity was categorised using the presence of sight threatening complications: corneal opacity involving the visual axis, cataract, ocular hypertension or glaucoma, macular oedema, or retinal or choroidal lesion causing irreversible negative impact on central acuity or visual field. Severity was also quantified using the degree of visual impairment caused by the complication drawing on WHO’s taxonomy for best corrected visual acuity (logMAR 0.0 to 0.2 logMAR=no vision impairment (VI), 0.2 to 0.48 logMAR=mild VI, 0.49 to 1.0 logMAR=moderate VI and level at which additional educational support is required, 1.01 LogMAR or worse=severe VI/ blindness). These acuity thresholds were also used to categorise uniocular visual loss.

The annual incidence (rate) of new diagnosis of childhood non-infectious uveitis was estimated using cases newly diagnosed during the 24-month period of 1 March 2021 to 28 February 2023. Relative incidence rates were reported by sex (female compared with male) and by different socioeconomic (most deprived quintile compared with those living in the other four less deprived areas) and ethnicity (White British background compared with White Other, Black British and Asian British) backgrounds. Denominator for UK children aged 0–18 were obtained from the UK ONS datasets for 2020/2021.^16^

Data were analysed using STATA statistical software (V.17.2, StataCorp LLC, College Station Texas) and R (V.4.0.2, R Foundation, Vienna, Austria). 95% CIs are reported, and a threshold of p<0.05 was used as a threshold for statistical significance.

## Results

**Figure 1:**
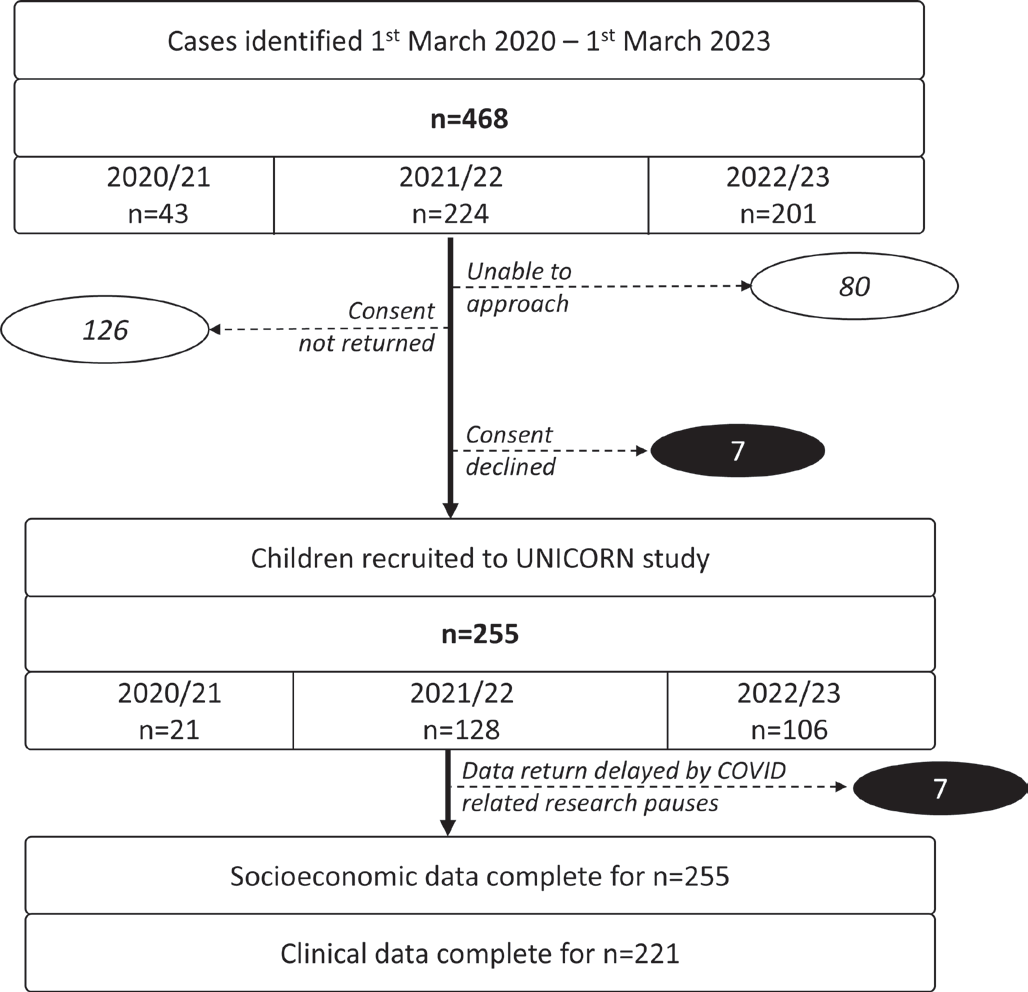
Uveitis in Childhood National Cohort Study participant flow from identification to data collection. Due to COVID-related research pauses, only four of the centres were returning case identification data at the start of the study (March 2020).

UNICORNS identified 468 newly diagnosed children/young people with non-infectious uveitis across 31 collaborating NHS hospitals between 1 March 2020 and 28 February 2023 (figure 1). These 31 hospitals form 89% of the reporting base.^6^ Of these 468 individuals, 255 (132 girls, 52.2%,) were recruited to the inception cohort study. Participation varied across collaborating sites, ranging from 0% to 100% of those approached (median 71%, IQR 50%–80%).

### Cohort characteristics at diagnosis

#### Socio-demographic

The characteristics of children recruited to the UNICORNS are reported in table 1. Children resident in the most deprived areas in the UK (lowest quintile, ie, lowest 20% of areas by deprivation) were over-represented within the population of children newly diagnosed with uveitis, comprising 28% of those within the cohort. Children from non-White ethnic backgrounds were also over-represented, comprising 31.4% of the cohort. Children from ‘White other’ (including those self-describing as being White Central or White Eastern European, n=19) comprised 28/255, 11% of the cohort.

**Table 1:** Sociodemographic characteristics within the UNICORNS

	Total n=255
Gender:	
Female	132, 51.8%
Self-reported ethnicity	
White British*	148, 58.0%
White other	28, 11.0%
All Asian/Asian British†	34, 13.3%
Indian	6, 2.4%
Pakistani	15, 5.9%
Bangladeshi	9, 3.4%
Asian other	4, 4%
All Black/Black British‡	23, 9.1%
Black African	20, 7.8%
Black Caribbean	3, 1.2%
Mixed ethnicity	10, 3.9%
Other	6, 2.4%
Not reported by family	6, 2.4%
Residence IMD score	
Most deprived quintile	72, 28%
Age	
0–4 years	30, 13.6%
5–10 years	82, 37.1%
11–15 years	96, 43.4%
16+years	13, 5.9%

a,b,cComparative population level frequency data on ethnicity of UK children.

* White British 73% (significant difference from study cohort, χ2 29.1, p<0.001).

† Asian British 12%.

‡ Black British 5% (significant difference from study cohort, χ2 9.0 p<0.01).

IMD, index of multiple deprivation.

**Figure 2:**
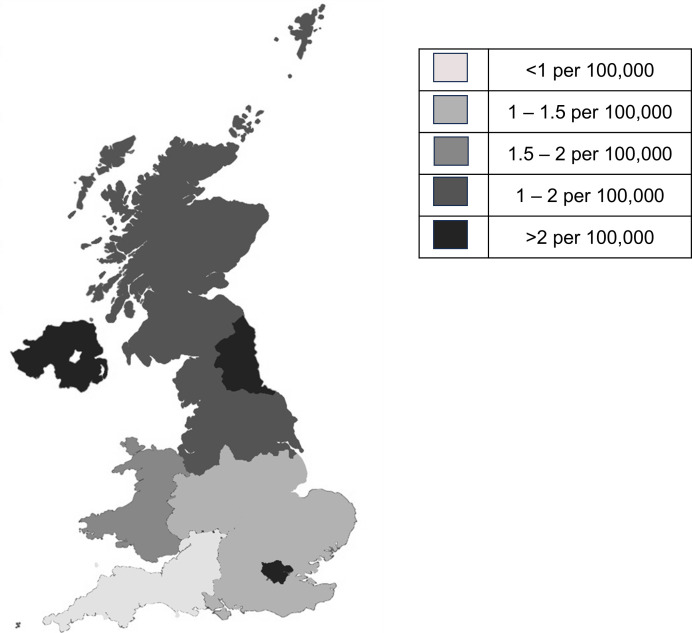
Geographic distribution of cases by incidence per population aged <18 years.

Children ranged from 1.7 to 17.7 years in age at diagnosis, with a median of 10.1 years. There was a bimodal pattern to age at diagnosis, with a peak at 4–7 and another higher peak at 11–15 years (figure 2).

### Clinical characteristics

**Figure 3:**
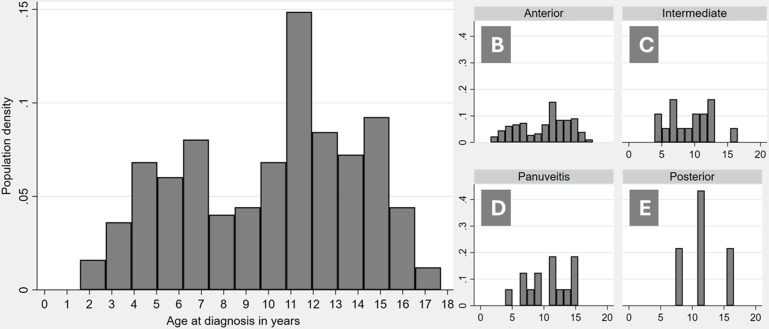
Age at diagnosis of uveitis for whole cohort (3A) and by uveitis type.

Anterior uveitis was the most common form of disease at presentation (170, 76.9%, table 2), followed by anterior and intermediate (17, 7.7%), intermediate (16, 7.2%), panuveitis (14, 6.3%) and posterior uveitis (4, 1.8%). Bilateral uveitis was seen in 144 children (65.2%), and of those who had unilateral disease at diagnosis, the right eye was involved in 41 (53.2%) and the left eye in 36 cases. Median age at onset for anterior uveitis was 9.9 years (range 1.7–17.7), intermediate 9.4 years (6.8–16.3), posterior 11.1 years (range 7.3–16.1) and panuveitis 11.3 years (range 4.3–16.0) (figure 3).

**Table 2:** Ocular and visual clinical characteristics of cohort at uveitis diagnosis

	Total n=221 children	Anterior n=187	Intermediate n=16	Posterior n=4	Panuveitis n=14
Vision in worse seeing eye					
Normal vision	123 (55.7%)	114	7	0	2
Mild loss	35 (15.8%)	25	3	1	6
Moderate loss	40 (18.1%)	31	3	2	4
Severe loss	23 (10%)	17	3	1	2
Bilateral vision impairment					
No visual impairment	184 (83.2%)	170	11	1	9
Mild visual impairment (VI)	20 (9.1%)	6	3	2	4
Moderate VI	8 (3.6%)	3	1	1	1
Severe VI/blind	9 (4.1%)	8	1	0	0
Inflammatory structural sequelae					
Visually significant cataract	5 (2.3%)	4	0	0	1
Macular oedema	15 (6.8%)	11	2	1	2
Ocular hypertension	51 (23.1%)	49	0	0	2
Glaucoma	0	0	0	0	0
Posterior synechiae (PS)*	74 (33.5%)	66	0	0	8
PS affecting 360 degrees of pupil	11 (5.0%)	9	0	0	2

Anterior includes ‘anterior/intermediate’.

* Posterior synechiae involving <180 degrees in 54/74 and >180 degrees of pupil in 20/74.

Associated non-ophthalmic immune-mediated disorders were diagnosed at or prior to uveitis diagnosis in 55 (24.9%, (online supplemental table S1) of the 221 children. These disorders comprised JIA (32, 14.5%), tubulointerstitial nephritis and uveitis (12, 4.7%), sarcoidosis, type I diabetes mellitus, inflammatory bowel disease and Behçet’s disease (each affecting ≤3 children).

Almost half of the children/young people in UNICORNS (44%) had some vision loss in at least one eye at diagnosis. Almost one in five had vision impairment (using WHO criteria and best corrected vision) (table 2), and 4% of children, most of whom had anterior uveitis, were severely visually impaired or blind at uveitis diagnosis.

### Incidence of childhood non-infectious uveitis in the UK

The annual minimum incidence of childhood non-infections uveitis from 1 March 2021 to 28 February 2023 was 1.89/100 000 (95% CI 1.72 to 2.07/100 000) using the nominator of all 425 cases identified between 1 March 2021 and 28 February 2023 across the 31 hospitals (of the 35 hospitals identified as potential participant identification centres). Regional incidences per 100 000 ranged from 3.13 in London and 2.13 per in Northern Ireland to 1.00 in the Northeast of England.

Overall, there was a higher relative risk of childhood non-infectious uveitis among those living in socioeconomic deprivation (relative risk (RR) 1.57, 95% CI 1.33 to 1.68, for children living in most deprived areas vs those from least deprived areas, online supplemental table S2) or children from ethnic minority backgrounds (RR 1.21, 95% CI 1.07 to 1.35, for children of Asian/Asian British background, and RR 1.76, 95% CI 1.56 to 1.87 for children from Black/Black British backgrounds, compared with children from White British backgrounds). There was no association of sex with disease risk (RR for male sex 0.99, 95% CI 0.98 to 1.03, when compared with female sex).

## Discussion

From a UK-wide inception cohort study, we demonstrate striking variations in risk of non-infectious childhood onset uveitis by demographic and socioeconomic factors, with those living in deprivation or those from ethnic minority backgrounds being at increased risk. Some degree of vision loss at diagnosis is common, with one in five children having vision that would fall below driving thresholds, and 8% having visual loss sufficient to mandate special educational provision or Government funded support. The relative contribution of JIA-associated uveitis was lower than anticipated (based on the prior literature), while the proportion of children with tubulointerstitial nephritis and uveitis was higher than expected. An asymmetric bimodal distribution of age at disease onset was noted, with a peak in adolescence. The overall incidence was higher than anticipated, based on prior studies.

JIA has dominated the childhood uveitis evidence base. This is partly due to the high burden of disease among affected children, but likely also reflects that much of the work in the field has been driven by paediatric rheumatology. The relatively low contribution of JIA to UNICORNS is unlikely to be due to a particular failure to recruit those children into the study, as supported by recent examination of participation patterns at the largest recruiting site.^17^ It is possible that over time, JIA will be identified/diagnosed in some of the UNICORNS cohort, since in a small proportion of children with JIA (estimated at 5–7%), the eye involvement presents before arthropathy manifests.^18^ It is more plausible that there has been a true decline in incidence of JIA-uveitis over time through earlier use of steroid sparing DMARDs in JIA.^11^ These agents are known to confer some protection against the development of uveitis,^5 19^ although this protection may lapse with their discontinuation in adulthood.^20^

Notably, we found that the diagnosis of uveitis is typically made in adolescence, an especially critical time during which profound physiological, social and mental health vulnerabilities can emerge,^21^ while simultaneously young people are also expected to take effective control of the organisation of their own healthcare.^22^ Superimposed on this is the challenge that the treatment of their uveitis—as a chronic but unpredictable disorder—requires an iterative ‘trial and error’ approach which therefore also requires maximum concordance from the patient. Most children within the UK with rare disorders such as uveitis are managed at specialist paediatric centres.^6^ Care is provided in child centred environments, in locations and with teams very different to those they will go on to experience following their transfer to adult care centres. The population-based data provided here helps to frame uveitis as a disease of adolescence, putting greater focus on the care gaps and the broader medical and psychosocial needs of affected individuals and their families.

To date, understanding of the epidemiology of uveitis in the UK has drawn on research that did not report on ethnic background or socio-economic status^4 8 23^ or has drawn on populations with JIA, in which uveitis occurs more commonly in children from White British/North and West European backgrounds.^24 25^ UNICORNS’ uncovering of the increased risk to and over-representation of individuals from underserved groups has wide implications. It is relevant to clinical practice and policy by contextualising care in the likely wider social needs of patients, for example, potentially differentially higher impact of the time lost from work or from school on the financial present and financial futures of children, young people and their families.^26^ These have been brought into sharper focus by the stark inequalities in access, experiences and outcomes of patients during the global COVID-19 pandemic, which exacerbated existing health inequities across and within nations. Prior to the pandemic, within the UK, there was a growing evidence base on health inequities within paediatric ophthalmology. For example, children from underserved populations were less likely to have families able to recognise the signs of visual disorders^27^ and were less likely to have access to healthcare innovations.^28^ These families were more likely to have children with blinding ocular disorders per se,^27^ and affected children were more likely to have adverse outcomes following interventions.^29^ Additionally, these families are less likely to take part in medical research.^30^ A key strength of UNICORNS is its potential to enable important insights into differential outcomes by socioeconomic and demographic grouping in due course.

The annual incidence of non-infectious uveitis reported is not directly comparable with prior reports due to differences in study methods. Nevertheless, it is higher than expected based on a national study reporting the annual incidence of any non-JIA-associated uveitis in childhood of 0.59 per 100 000 which was conducted using active surveillance through the British Ophthalmological Surveillance Unit.^31^ By contrast, the incidence reported here is lower than that identified across four hospitals in South and East England over the period from 1995 to 2001, reported as an annual incidence of 4.85 per 100 000 for all non-infectious childhood uveitis (2.3 per 100 000 for non-JIA uveitis).^4^ This is similar to the 1980–1982 incidence of 3.8/100 000 for non-infectious childhood uveitis (JIA uveitis incidence 1.6/100 000) reported by a study in Finland,^32^ but higher than the 2.5/100 000 reported for Northern Ireland across 2011–2015.^7^ Four UK centres managing eligible children were unable to take part in UNICORNs. Assuming similar proportions of identified cases across those four centres, the projected estimated incidence across the whole reporting base would be 2.18/100,000 (95% CI 1.93 to 2.46/100 000). It is not possible to make firm conclusions about temporal trends in incidence overall.

It is possible that the UNICORNS derived incidence is an overestimation of disease, as the study window used for incidence analyses opened at a time when eye care was becoming more accessible, that is, during the restoration of services following pandemic-related disruptions in UK health services.^33^ Consequently, delays in the presentation of cases may have resulted in a high apparent disease incidence during the study period. This challenge was, however, addressed using a 2-year ascertainment window.

Regional variability in disease incidence for surveillance studies may reflect ‘true’ disease incidence across a study reporting base but may also reflect the clinical research environments across that base. This study took place at a time of significant national disturbance, with the pandemic resulting in recruitment halts or pauses across the network. Notably, higher incidence, possibly more reflective of the ‘true’ disease incidence, were reported at nation states where care for children newly diagnosed was centralised to one NHS trust (Northern Ireland) or regions where case reporting was supported by the clinical role of the UNICORNS senior investigator (London). There is also the challenge of identifying whether there were other potential cases of childhood uveitis beyond the 35 identified clinical centres. Nevertheless, due to the wide involvement of clinicians in all four member states of the UK, the information provided by the UNICORNS cohort on the characteristics and distribution of disease should be nationally representative.

The scale and reach of UNICORNS also permitted insights relating to specific entities. Tubulointerstitial uveitis and nephritis are rare disorders whose incidence appears to have increased during and following the global COVID-19 pandemic.^34^ This appears to be reflected in our study. The single case of Tubulointerstitial Nephritis and Uveitus (TINU) identified during a 1-year ascertainment window (2014/2015)^8^ contrasts with the 12 tissue biopsy supported cases within UNICORNS. A SARS-CoV-2-related causal relationship with TINU has been posited, although an alternative explanation is greater recognition of TINU as a disease entity.^35^ UNICORNS is well placed to provide further natural history data on outcomes for those children diagnosed with TINU during and following the pandemic, providing a comparative group for the historical reports on outcomes for this still poorly understood disorder.

In conclusion, the findings reported here from UNICORNS indicate a need to reconsider how best to deliver paediatric rheumatological and eye care that meets the needs of young people with uveitis. Non-JIA uveitis appears to be growing in incidence, with a resultant need to ensure that multidisciplinary care structures have appropriate involvement of rheumatological and child health professionals alongside eye care services. There is also a need to improve the identification of children at risk prior to disease onset to avoid the visual loss too often present at diagnosis. The peak incidence during adolescence brings the need to improve care pathways for young people with this complex disorder. There appear to be socioeconomic drivers of disease risk, which are worthy of future exploration. Future evidence from this nationally representative and diverse cohort on care pathways, outcomes (ocular, systemic and quality of life) and the determinants of those outcomes will provide the currently unavailable data necessary to improve and develop services and inform clinical practice for children with non-infectious uveitis.

## Supplementary material

10.1136/bjo-2024-326674 online supplemental file 1

10.1136/bjo-2024-326674 online supplemental file 2

## Data Availability

Data are available upon reasonable request.
