# Supplementary material for: Incidence, sociodemographic and presenting clinical features of childhood non-infectious uveitis: findings from the UK national inception cohort study
Source: Br J Ophthalmol. 2025 Feb 11;109(8):e326674. doi: 10.1136/bjo-2024-326674 (PMC12320610; doi:10.1136/bjo-2024-326674)
Supplement: online supplemental file 1 [file bjo-109-8-s001.pdf]

Supplemental Table S1. Non ocular diagnoses or symptoms at presentation

		Total n=221	Anterior n=187	Intermediate n=16	Posterior n=4	Panuveitis n=14
	JIA	32 (14.5%)	30	0	0	2
	TINU	12 (5.4%)	12	0	0	0
	Other immune-mediated disease	11 (5.0%)	8	0	0	3
	Initially isolated uveitis	166 (75.1%)	137	16	0	9
	*No systemic complaints**	114 (51.6%)	94	11	0	7
	Musculoskeletal symptoms	16 (7.2%)	15	0	0	0
	Skin	6 (2.7%)	6	0	0	0
	Gastrointestinal	12 (5.4%)	10	2	0	0
	Fever	2 (<1%)	2	0	0	2

**In children with initially isolated uveitis (ie, absence of pre-existing systemic disorder, and absence of disorder diagnosed at time of uveitis diagnosis)*
