# Supplementary material for: Incidence, sociodemographic and presenting clinical features of childhood non-infectious uveitis: findings from the UK national inception cohort study
Source: Br J Ophthalmol. 2025 Feb 11;109(8):e326674. doi: 10.1136/bjo-2024-326674 (PMC12320610; doi:10.1136/bjo-2024-326674)
Supplement: online supplemental file 2 [file bjo-109-8-s002.pdf]

Supplemental Table S2. Relative incidence rates of childhood non-infectious uveitis

		Relative incidence
Ethnicity		
	White British	1
	All Asian / Asian British	1.21 (95% CI 1.07 – 1.35, $p=0.06$)
	All Black / Black British	1.76 (95% CI 1.56 – 1.87, $p<0.01$)
Sex		
	Female	1
	Male	0.99 (95% CI 0.98 – 1.03, $p=0.7$)
Residence deprivation score		
	Least deprived areas	1
	Most deprived areas (quintile)	1.57 (95% CI 1.33 – 1.68, $p<0.01$)
